# Chronic traumatic encephalopathy and aging-related tau astrogliopathy in community-dwelling older persons with and without moderate-to-severe traumatic brain injury

**DOI:** 10.1093/jnen/nlae007

**Published:** 2024-01-31

**Authors:** Sonal Agrawal, Sue E Leurgans, Lisa L Barnes, Kristen Dams-O’Connor, Jesse Mez, David A Bennett, Julie A Schneider

**Affiliations:** Rush Alzheimer’s Disease Center, Rush University Medical Center, Chicago, Illinois, USA; Department of Pathology, Rush University Medical Center, Chicago, Illinois, USA; Rush Alzheimer’s Disease Center, Rush University Medical Center, Chicago, Illinois, USA; Department of Neurological Sciences, Rush University Medical Center, Chicago, Illinois, USA; Rush Alzheimer’s Disease Center, Rush University Medical Center, Chicago, Illinois, USA; Department of Neurological Sciences, Rush University Medical Center, Chicago, Illinois, USA; Department of Behavioral Sciences, Rush University Medical Center, Chicago, Illinois, USA; Department of Rehabilitation and Human Performance, Mt Sinai School of Medicine, New York, New York, USA; Department of Neurology, Mt Sinai School of Medicine, New York, New York, USA; Boston University Alzheimer’s Disease Research Center, Boston University Chobanian & Avedisian School of Medicine, Boston, Massachusetts, USA; Boston University Chronic Traumatic Encephalopathy Center, Boston University Chobanian & Avedisian School of Medicine, Boston, Massachusetts, USA; Rush Alzheimer’s Disease Center, Rush University Medical Center, Chicago, Illinois, USA; Department of Neurological Sciences, Rush University Medical Center, Chicago, Illinois, USA; Rush Alzheimer’s Disease Center, Rush University Medical Center, Chicago, Illinois, USA; Department of Pathology, Rush University Medical Center, Chicago, Illinois, USA; Department of Neurological Sciences, Rush University Medical Center, Chicago, Illinois, USA

**Keywords:** Amyloid-β, ARTAG, Community-based study, CTE, Tau, Traumatic brain injury

## Abstract

This study examined the frequency of chronic traumatic encephalopathy-neuropathologic change (CTE-NC) and aging-related tau astrogliopathy (ARTAG) in community-dwelling older adults and tested the hypothesis that these tau pathologies are associated with a history of moderate-to-severe traumatic brain injury (msTBI), defined as a TBI with loss of consciousness >30 minutes. We evaluated CTE-NC, ARTAG, and Alzheimer disease pathologies in 94 participants with msTBI and 94 participants without TBI matched by age, sex, education, and dementia status TBI from the Rush community-based cohorts. Six (3%) of brains showed the pathognomonic lesion of CTE-NC; only 3 of these had a history of msTBI. In contrast, ARTAG was common in older brains (gray matter ARTAG = 77%; white matter ARTAG = 54%; subpial ARTAG = 51%); there were no differences in severity, type, or distribution of ARTAG pathology with respect to history of msTBI. Furthermore, those with msTBI did not have higher levels of PHF-tau tangles density but had higher levels of amyloid-β load (Estimate = 0.339, SE = 0.164, p = 0.040). These findings suggest that CTE-NC is infrequent while ARTAG is common in the community and that both pathologies are unrelated to msTBI. The association of msTBI with amyloid-β, rather than with tauopathies suggests differential mechanisms of neurodegeneration in msTBI.

## INTRODUCTION

Traumatic brain injury (TBI) is a major public health problem and a leading cause of mortality and morbidity in the United States and globally (1). Individuals who have experienced moderate-to-severe TBIs (msTBIs) often encounter ongoing difficulties in resuming work, maintaining social relationships, and engaging in typical daily activities (2, 3). Moreover, msTBI is associated with a higher risk of experiencing health issues, cognitive impairment, and neurodegenerative diseases later in life (3–9).

Chronic traumatic encephalopathy (CTE) is a progressive neurodegenerative disease that can only be definitively diagnosed postmortem through neuropathologic examination. Studies have consistently shown a strong, dose-response relationship between the amount of contact sport play, a proxy for repetitive head impacts (RHIs), and the presence of CTE-neuropathologic change (CTE-NC) (10–15). However, findings of msTBI in relation to CTE-NC are more mixed (4, 6, 16–18), with most studies conducted in clinic-based and autopsy-based samples. These studies have shown inconsistent relationships, are more susceptible to selection bias and may not generalize to the community. Thus, it remains important to understand the frequency of CTE-NC within community settings in those with and without msTBI; and to explore the potential link between msTBI and CTE-NC.

The current consensus criteria for neuropathological identification of CTE-NC requires the accumulation of perivascular p-tau neuronal aggregates at the depth of a cortical sulcus with and without age-related tau astrogliopathy (ARTAG), primarily represented by subpial thorn-shaped astrocytes (TSAs) as supporting features (19). The presence of subpial TSAs in CTE-NC individuals suggests that its potential involvement in TBI pathophysiology (20). Limited research has been conducted on the association between TBI and ARTAG in community-dwelling people (21) and even less is known about the association of msTBI and ARTAG outcomes. Furthermore, the frequency of CTE-NC, CTE-NC supporting features, and ARTAG pathologies have not been systematically investigated; therefore, a better understanding of these tauopathies in community-based subjects could potentially help us understand the relevance of these brain pathologies to clinical outcomes after msTBI exposure.

In the current study, we examined the frequency and association of CTE-NC, CTE-NC supporting features, and ARTAG in community dwelling older persons with msTBI compared to those without TBI. We also examined the relationship of msTBI with overall PHF-tau tangles and total phosphorylated tau protein in the brains of older persons.

## MATERIALS AND METHODS

### Study participants

All subjects were participants in 1 of 3 ongoing epidemiologic longitudinal community-based cohort studies of aging: the Religious Orders Study (ROS), the Rush Memory and Aging Project (MAP), or the Minority Aging Research Study (MARS) (22, 23). Briefly, study participants were community-dwelling older people without known dementia at the time of enrollment who agreed to undergo annual clinical evaluation and interview. Brain donation is required in ROS and MAP studies, while brain donation is optional in MARS. All 3 studies were approved by the Institutional Review Board of Rush University Medical Center. We included 1419 deceased and autopsied ROS-MAP-MARS participants who had available antemortem data on the history of TBI and had available neuropathology data on major brain pathology including AD pathology. To focus the study of CTE-NC and ARTAG on people with msTBI exposure, defined as a TBI with loss of consciousness (LOC) >30 minutes, we excluded participants with TBI with LOC <30 minutes, those with TBI with unknown LOC, or those with TBI without LOC from this study, leaving a total of 981 participants. Among these 981 participants, 94 had a history of msTBI exposure. Each of the 94 was then matched one-to-one with an unexposed participant by age at death, sex, years of education, and dementia status proximate to death. For example, for each female participant with dementia and msTBI, we identified the eligible non-TBI female participant with dementia whose age at death and years of education were closest to the index participants. Closeness was quantified by the Mahalanobis distance, which is a multivariate distance that considers the correlations of (in this case) age at death and education. The matched participant was then removed from the pool of eligible participants. This matching process resulted in a total of 188 participants for the analyses. The mean age at the time of enrollment was 72.76 years (SD = 7.94; range from 63 to 98 years). The group exposed to msTBI had an average age at death was 88.2 (SD = 8.15) years and 16.8 (SD = 4.08) years of education on average. The matched group of persons not exposed to TBI had an average age at death of 88.4 (SD = 7.83) years and 16.9 (SD = 3.95) years of education on average. In both groups, 46% were male, and 45% had dementia proximate to death.

### TBI assessment

TBI information was obtained via self-report upon enrollment in the study by using the two-item questions, first, “Have you ever sustained a brain injury?” and second, “if yes, whether it resulted in the LOC?” and at every follow-up visit using the same question “Since your last study visit, have you had a head injury” and “if yes then have you lost consciousness because of head injury.” If a head injury w/LOC was reported, participants were then asked to determine the duration of LOC. Information regarding sporting careers, military services, or physical abuse was not collected. The classification of msTBI was determined solely by considering the duration of LOC, which is one of several criteria used to denote severity of TBI. Other methods like the Glasgow Coma Scale, alteration of mental status, and the duration of posttraumatic amnesia were not employed in this determination (24, 25). Finally, the participants were categorized into msTBI group (TBI with LOC greater than 30 minutes) and control group (no TBI exposure).

### Neuropathology assessment

The median postmortem interval was 6.53 hours (IQR = 4.9–10.5 hours). Following autopsy and brain removal, one hemisphere was dissected into 1-cm coronal slabs and frozen in 80°C for tissue distribution. The contralateral hemisphere underwent fixation in 4% paraformaldehyde for a minimum of 48–72 hours before being cut into 1-cm coronal slabs (26, 27). We collected multiple brain regions, including the midfrontal, superior frontal, middle temporal, inferior orbital frontal, anterior cingulate, entorhinal, anterior temporal, inferior parietal, and occipital cortices, as well as the amygdala, hippocampus, basal ganglia, substantia nigra, and cerebellum, for various pathology data collection from the paraformaldehyde-fixed tissue, as detailed below.

### CTE-NC assessment

Tissue blocks from the midfrontal, middle temporal, superior frontal, and anterior temporal tip cortices were sampled from the fixed hemisphere for CTE-NC assessment. At the time of blocking, we tried to include cortical tissue from as many depths of sulci as possible. Paraffin-embedded sections were cut at 6-μm thickness and stained with an antibody specific to phosphorylated tau (AT8; 1:2,000, Thermo Fisher Scientific, Rockford, IL) to identify hyperphosphorylated (p)-tau. p-tau neurons around the blood vessel were evaluated in both the depth of sulcus and the gyral crest from each cortical region. We used the second NINDS/NIBIB consensus criteria to categorize CTE-NC (19). According to the second consensus criteria, the participant was considered to have CTE-NC lesion if any of the cortical regions contained p-tau neuronal aggregates around small blood vessels in the depths of sulci. Cases were screened by a neuropathologist (J.A.S.) and trained neuroscientist (S.A.). Select cases and those with high Braak stages (IV–VI) were rereviewed with the senior neuropathologist (J.A.S.) for the presence and number of CTE-NC lesions. The number of CTE-NC lesions was counted in each participant and categorized as none [0], single [1], or multiple [>1]. In older persons, identifying perivascular tau tangles can be difficult because of AD related tau pathology in the vicinity of vessels. For our study, we empirically operationalized circumferential perivascular tau by semiquantitatively assessing the percentage of the blood vessel circumference distinctly occupied by p-tau tangles. We used the following empiric criteria: mild when p-tau tangles pathology occupied up to 50% of the circumference, moderate when it occupied 50%–75% of the circumference, and severe when it occupied more than 75% of the circumference. Both examiners reviewed all cases with evidence of tau tangles near to blood vessels. If there were multiple CTE-NC lesions, the blood vessel with the greatest circumference occupied by p-tau tangles was used for grading the severity of the whole brain. This protocol was operationalized after reviewing numerous tau-stained slides in a cohort of older individuals, many with AD pathology. This methodology was empirically derived with the goal of enhancing detection and minimizing misclassification of CTE in the presence of varying levels of AD pathology. Participants with moderate-to-severe perivascular neuronal tau tangle pathology in the depth of any cortical sulci were considered “definite” CTE-NC and the participants with mild perivascular neuronal tau tangle pathology in the depths of any sulci were classified as “possible” CTE-NC. It is important to note these labels do not indicate the previously published stages of CTE-NC pathology (19) but rather the likelihood that the brain exhibited CTE-NC. In addition, we sampled dentate nucleus of the cerebellum and assessed neurofibrillary tangles as this is described earlier as positive in the advanced CTE-NC cases (19, 28).

### CTE-NC-supporting pathologic features

The same AT8 stained slides from the midfrontal, middle temporal, superior frontal, and anterior temporal tip cortices described above were used for the assessment of CTE-NC supporting pathologic features. Supporting pathologic features include characteristics of neuronal tau pathology and specific astrocytic tau pathologies (some also considered under the rubric of ARTAG). Specifically, CTE-NC-related supporting features include pattern and distribution of p-tau neuronal tangles and presence of subpial TSAs in any deep sulci. In brief, the pattern of p-tau tangles was recorded as patchy or continuous while the distribution was recorded based on whether p-tau is predominantly located either in superficial cortical layers (layers II/III) or deep cortical layers (layers IV/V) of the cerebral cortex. Participant was considered to have patchy distribution and superficial predilection of p-tau tangles if any of the cortical regions contained both cellular components. The subpial TSAs in each cortical region were recorded as present or absent in both depth of sulci and gyri as present or absent. The participant was classified as having subpial TSAs in sulci if any of the 4 cortical regions had subpial TSAs at the depth of a sulcus. We also conducted a separate assessment for the presence and the severity of perivascular p-tau astrocytes aggregates from the 4 cortical regions described above. These glial aggregates were scored separately from the CTE-NC lesion but in some cases perivascular p-tau astrocytes co-occurred with the CTE lesions. It is important to note that both subpial TSAs and perivascular p-tau astrocytic aggregates are also a component of ARTAG.

### ARTAG assessment

ARTAG pathology including TSAs and granular fuzzy astrocytes (GFAs) was assessed from the 6 µm AT8 immunostained sections of the amygdala, anterior temporal tip, and superior frontal cortex. Each brain region was reviewed for TSA and GFA pathologies from 3 anatomical locations consisting of the gray matter, white matter, and subpial cortex (29). In brief, a graticule was used to count the total number of each ARTAG measure in a 0.25-mm^2^ highest density area (×20 magnification) from gray matter, white matter, and subpial anatomical locations of 3 brain regions. Each ARTAG measure was separately rated from 0 (none) to 5 (severe) on a 6-point scale. The overall brain ARTAG burden was defined as the highest semiquantitative measure of ARTAG burden across the anatomical location of 3 brain regions. Along with this, overall gray matter ARTAG, overall white matter ARTAG, and overall subpial ARTAG were defined as the highest semiquantitative measure of ARTAG burden across the 3 brain regions for specific anatomical locations.

We also defined regional amygdala and neocortical gray matter, white matter, and subpial ARTAG. For example: neocortical gray matter ARTAG was defined as the maximum of the semiquantitative measures of ARTAG burden from gray matter location of the 2 neocortical brain regions. For descriptive statistics, a dichotomous variable present and absent was used for overall brain ARTAG as well as for overall and regional amygdala and neocortical gray matter, white matter, and subpial. For statistical models, a semiquantitative 3-level grading system (0–2) was created for overall and regional amygdala and neocortical gray matter, white matter, and subpial ARTAG burden where 0 (none, no TSAs and GFAs observed), 1 (mild); 1–5 TSAs and GFAs, and 2 (moderate-severe; 6 or more TSAs and GFAs). Because there was a minimal ARTAG burden in the white matter and subpial locations of the neocortical regions, we used a dichotomous measure (present or absent) for neocortical gray matter, white matter, and subpial ARTAG in statistical models. Subpial neocortical ARTAG is also considered under CTE-NC supporting features.

### Alzheimer disease pathology

Modified Bielschowsky silver-stained 6-µm sections from the middle temporal, entorhinal, midfrontal, and inferior parietal cortices and hippocampus were used to count neuritic plaques and neurofibrillary tangles to classify the Consortium to Establish a Registry for Alzheimer’s Disease (CERAD) system (30) and Braak staging (31), respectively. Thal phase was determined from 7 brain regions: midfrontal, middle temporal, and inferior parietal cortices, hippocampus, basal ganglia, substantia nigra, and cerebellum using immunostaining with a monoclonal antibody against amyloid-β (4G8; dilution: 1:9000; Covance Labs, Madison, WI) (32). Finally, 4 levels of AD neuropathologic change including “Not,” “Low,” “Intermediate,” or “High” ADNC was assessed using National Institute on Aging-Alzheimer’s Association criteria (32). For analyses, a dichotomized version of the ADNC was used where participants with intermediate or high AD neuropathologic change indicate a pathologic diagnosis of AD and participants with not or low AD neuropathologic change indicate no/low pathologic diagnosis of AD. In addition, diffuse and neuritic plaques burden was determined by microscopic examination of silver-stained slides from 5 regions: midfrontal cortex, middle temporal cortex, inferior parietal cortex, entorhinal cortex, and hippocampus. The count of each region is scaled by dividing by the corresponding standard deviation. The 5 scaled regional measures are then averaged to obtain a summary measure for diffuse and neuritic plaques burden (33).

PHF-tau tangles were identified by an antibody specific to phosphorylated tau, AT8 on 20-μm sections from 8 brain regions, including midfrontal, superior frontal, anterior cingulate, entorhinal, inferior temporal, inferior parietal, ad calcarine cortices, and CA1/subiculum subfield of the hippocampus. A systematic random sampling and optical fractionator probe including a counting frame of 150 by 150 µm and sampling grid of 410 by 410 µm were used to obtain an estimated quantitative measure for the total number of neurofibrillary tangle cell counts within a defined area, which was then calculated as a density measure per square millimeter (27). Similarly, amyloid-β was identified from the same 8 brain regions mentioned above by any of the 3 antibodies using 3 monoclonal antibodies against Aβ (4G8; dilution: 1:9000; Covance Labs, 6F/3D; dilution: 1:50; Dako North America, Inc., Carpinteria, CA, and 10D5; dilution: 1:600; Elan Pharmaceuticals, San Francisco, CA). The systematic random sampling grid of 1200 by 900 µm within the Stereo Investigator software was applied to each region. A percentage of amyloid positivity was calculated using a custom positive-pixel algorithm. For analyses, the percentage of amyloid positivity was averaged across regions (27). Because both tangles and amyloid-β measures were right-skewed, both measures were square root transformed and averaged across regions to obtain cortical amyloid-β load and PHF-tangle density.

### Other age-related non-AD pathologies

The presence of neocortical LBs was assessed using immunohistochemistry with α-synuclein (Zymed LB 509; 1:50; pSyn, 1:20 000; Wako Chemicals) from 7 brain regions and analyzed as a dichotomous variable based on the presence in any of the assessed neocortical regions in the analyses (34). LATE pathology was determined by immunohistochemistry using a rat phosphorylated monoclonal TAR5P-1D3 (pS409/410; 1:100, Ascenion, Munich, Germany) TDP-43 antibody. LATE-NC distribution was divided into 3 stages. In stage 1, LATE-NC was localized to the amygdala; in stage 2, there was an extension to the hippocampus or entorhinal cortex while in stage 3, there was an extension to neocortical areas. For analyses, LATE-NC distribution was dichotomized into low (none and stage 1) and high (stages 2–3) (26). Hippocampal sclerosis (HS) was evaluated unilaterally in a coronal section of the midhippocampus and graded as absent or present based on severe neuronal loss and gliosis in CA1 and/or subiculum sector (26).

### Targeted proteomics: selected reaction monitoring

Tau phosphopeptides abundance was quantified using quantitative selected reaction monitoring proteomics from frozen dorsolateral prefrontal tissue samples (gray matter) (BA 9) as described elsewhere (35). We assessed the status of the following tau domains: AT8, AT100, 12E8, 77G7, and PHF-1 by quantifying the phosphorylation at the S202, T217, S262, S305, and S404 residues, respectively.

### Other covariates

Sex, race, ethnicity, and years of education were obtained through self-reports at baseline. APOE genotyping was evaluated by sequencing rs7412 (codon 158) and rs429358 (codon 112) at exon 4 of the APOE gene as described elsewhere (36). The final clinical diagnosis of Alzheimer dementia was made by a board-certified neurologist using all available clinical data blinded to the neuropathologic evaluation and participants with one impaired domains were considered to have mild cognitive impairment (MCI), participants with probable AD dementia (AD+no other cause of cognitive impairment), possible AD dementia (AD+other cause contributing to cognitive impairment) or other (other primary cause of dementia) were considered to have dementia, and participants without cognitive impairment, i.e. those without dementia or MCI, were considered as no cognitive impairment (NCI) (37). Vascular risk factors including hypertension and diabetes were classified based on self-report and medial review and smoking history was reported at enrollment (38, 39). Vascular diseases such as myocardial infarction and claudication were defined by self-report and stroke was defined by self-reported medical history and by the physician on the basis of a uniform structured neurological examination (39).

### Statistical analysis

McNemar tests were used to compare the fractions with CTE-NC diagnostic pathology and perivascular astrocytic tau pathology between the msTBI and matched control groups. Chi-square tests and logistic regression analyses were performed to examine the association between msTBI and CTE-NC supporting features. Wilcoxon signed-rank tests were used to compare the ARTAG pathology among msTBI and matched control groups. Other age-related pathologies including amyloid-β load, PHF-tau tangles density, neocortical LBs, LATE-NC, and HS, were compared between decedents with msTBI exposure and those without reported msTBI exposure using the Wilcoxon-Mann-Whitney tests or chi-square tests. Exact 95% confidence limits are supplied for proportions. Linear regression analyses were performed to examine the association of msTBI exposure with amyloid-β load and PHF-tau tangles density and separately with phosphorylated tau peptides, while logistic regression analyses were performed to examine the association of msTBI exposure with pathologic diagnosis of AD, ARTAG, neocortical LBs, LATE-NC, and HS adjusted for demographics. All analyses were programmed in SAS/STAT version 9.4 (SAS Institute Inc., SAS/STAT 14.1 User’s Guide, Cary, NC). A nominal threshold of p < 0.05 was used for statistical significance.

## RESULTS

One hundred eighty-eight participants, 94 with and 94 without msTBI, were included in this study. Overall, the mean age-at-death was 88.32 (SD = 7.97) years, 46% were male, 176 (93%) were non-Latino whites, 5 (3%) were non-Latino black, and 7 (4%) were Latinos. One-fourth of the participants were APOEε4 carriers, and nearly half of the participants (45%) had dementia proximate to death. Demographic and clinical, and pathological characteristics for the msTBI and non-TBI groups are summarized in Table 1.

**Table 1.** Characteristics of msTBI and unexposed TBI participants

Characteristics	Total (n = 188)	No TBI (n = 94)	msTBI (n = 94)	p-values
Demographic				
Age-at-death, mean (SD)	88.32 (7.97)	88.4 (7.83)	88.2 (8.15)	NA*
Male, n (%)	86 (45.7%)	43 (45.7%)	43 (45.7%)	NA*
Education, mean (SD)	16.92 (4)	16.9 (3.95)	16.89 (4.08)	NA*
Non-Latino Whites, n (%)	176 (93.62)	87 (92.5%)	89 (92.7%)	0.550
Clinical				
Dementia, n (%)	84 (44.7%)	42 (44.7%)	42 (44.7%)	NA*
Neuropathologic				
CTE-NC measures				
CTE-NC lesion, n (%)	6 (3.1%)	3 (3.1%)	3 (3.1%)	NA^†^
Definite CTE-NC, n (%)	2 (1.0%)	1 (1.0%)	1 (1.0%)	NA^†^
Possible CTE-NC, n (%)	4 (2.1%)	2 (2.1%)	2 (2.1%)	NA^†^
Perivascular astrocytic p-tau, any location, n (%)	8 (4.2%)	4 (4.2%)	4 (4.3%)	NA^†^
Perivascular astrocytic p-tau in gyri, n (%)	4 (2.1%)	3 (3.1%)	1 (1.0%)	NA^†^
Perivascular astrocytic p-tau in depth of sulci, n (%)	8 (4.2%)	4 (4.2%)	4 (4.2%)	NA^†^
Subpial TSAs, any location, n (%)	33 (17.5%)	15 (15.9%)	18 (19.1%)	0.565
Subpial TSAs at sulci, n (%)	22 (11.2%)	11 (11.7%)	11 (11.7%)	1
Subpial TSAs at gyri, n (%)	25 (13.3%)	10 (10.6%)	15 (15.9%)	0.282
p-tau tangle distribution (patchy and superficial layer), n (%)	27 (14.3%)	12 (12.7%)	15 (15.9%)	0.532
ARTAG measures				
ARTAG burden, overall, n (%)	143 (80.3%)	72 (82.7%)	71 (78%)	0.426
GM ARTAG burden, overall, n (%)^‡^	137 (76.9%)	69 (79.3%)	68 (74.7%)	0.467
GM ARTAG burden, amygdala, n (%)^‡^	131 (73.6%)	66 (75.8%)	65 (71.4%)	0.502
GM ARTAG burden, neocortex, n (%)	101 (53.7%)	54 (57.4%)	47 (50%)	0.305
WM ARTAG burden, overall, n (%)^‡^	97 (54.4%)	49 (56.3%)	48 (52.7%)	0.632
WM ARTAG burden, amygdala, n (%)^‡^	97 (54.4%)	49 (56.3%)	48 (52.7%)	0.632
WM ARTAG burden, neocortex, n (%)^§^	29 (15.8%)	16 (17.3%)	13 (14.2%)	0.565
SP ARTAG burden, overall, n (%)^‡^	92 (51.6%)	49 (56.3%)	43 (47.2%)	0.226
SP ARTAG burden, amygdala, n (%)^‡^	88 (49.4%)	48 (55.1%)	40 (43.9%)	0.134
SP ARTAG burden, neocortex, n (%)^§^	27 (14.7%)	14 (15.2%)	13 (14.2%)	0.859
AD and non-AD neurodegenerative measures				
Overall amyloid-beta burden, mean (SD)	1.61 (1.2)	1.44 (1.2)	1.79 (1.2)	0.045
Overall PHF-tau tangles density, mean (SD)	1.52 (1.3)	1.68 (1.5)	1.35 (1.1)	0.204
Neuritic plaque burden, mean (SD)	0.79 (0.79)	0.78 (0.85)	0.79 (0.74)	0.931
Diffuse plaque burden, mean (SD)	0.77 (0.52)	0.74 (0.91)	0.80 (0.73)	0.618
Pathologic diagnosis of AD, n (%)	125 (66.8%)	61 (65.5%)	64 (68.1%)	0.717
Neocortical LBs, n (%)	19 (10.1%)	6 (6.3%)	13 (13.8%)	0.09
LATE-NC (≥stage 2), n (%)	52 (29.2%)	28 (31.8%)	24 (26.6%)	0.449
Hippocampal sclerosis, n (%)	16 (8.5%)	8 (8.5%)	8 (8.5%)	1

ADNC, Alzheimer disease neuropathologic change; ARTAG, aging-related tau astrogliopathy; CTE-NC, chronic traumatic encephalopathy neuropathological changes; GM, gray matter; LATE-NC, limbic-predominant age-related TDP-43 encephalopathy neuropathologic change; LBs, Lewy bodies; SD, standard deviation; SP, subpial; msTBI, moderate-to-severe traumatic brain injury; TSA, thorn-shaped astrocytes; WM, white matter.

* Significance tests and p-values are not appropriate because the msTBI group and no TBI groups were matched by age, sex, education, and dementia status.

† Significance tests and p-values are not appropriate because none of the CTE measures were compared between msTBI and no TBI groups due to infrequent pathologic findings.

‡ Measures not assessed for 10 participants.

§ Measures not assessed for 5 participants.

### Frequency of CTE-NC in community-dwelling subjects with and without msTBI

**Figure 1.**
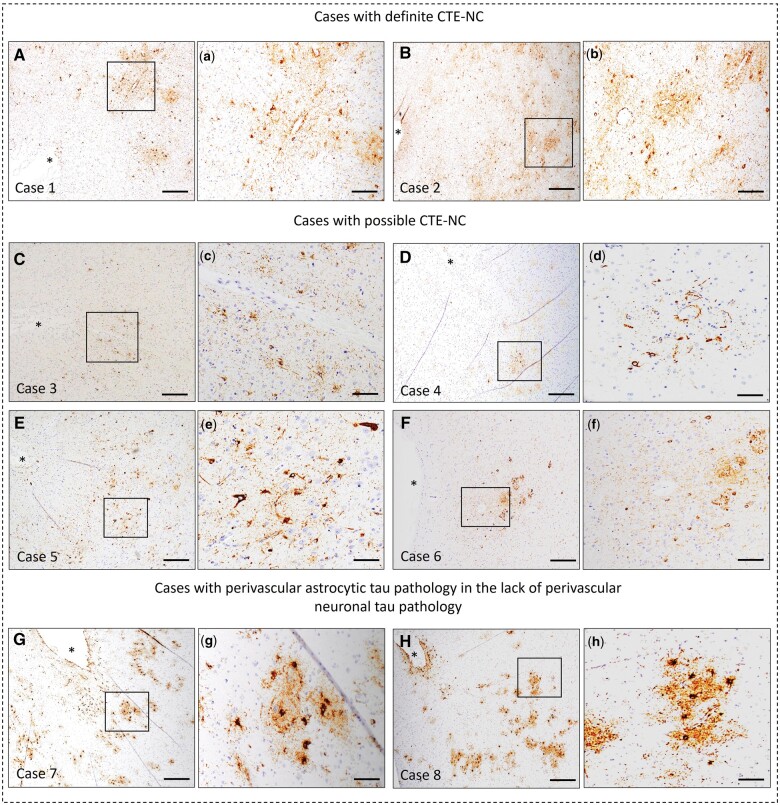
CTE-NC pathology in the brain. **(A, B)** Representative images of 2 participants with definite CTE pathology. **(C–F)** Images from 4 representative participants with possible CTE pathology. Black boxes indicate the area shown in the high-magnification image on the right **(a–f)**. Asterisk (*) indicates pial surface of sulcus. **(G, H)** Images from 2 representative participants show irregular deep gray matter perivascular astrocytic tau deposition at depth of sulci, but without a tau-immunopositive perivascular neuronal component. Black boxes indicate the location of the high-magnification image on the right **(g, h)**. A, B, D, G, H, superior frontal; C, midfrontal gyrus; E, F, middle temporal gyrus. Scale bars A–H = 200 μm; a–h = 100 μm.

Of these 188 decedents, CTE-NC lesions were recorded in 6 decedents (point estimate = 3.2%, Exact 95% confidence interval: 1.5%, 6.8%), of whom 4 decedents had p-tau neurons around blood vessels in the depth of a sulci only, 2 had in both gyri and sulci (Fig. 1). Four of 6 decedents had multiple CTE-NC lesions and 2 had single CTE-NC lesions. The total number of CTE-NC lesions in 4 participants with multiple CTE-NC lesions is presented in Table 2. In addition, none of these decedents had neurofibrillary tangles in the cerebellum, which would have indicated a more advanced CTE-NC case. Most of the decedents had CTE-NC lesions (n = 4/6) present in the frontal region, whereas 2 brains had CTE-NC lesions in the temporal cortex without the presence in the frontal cortex. We also examined the perivascular neuronal tau severity. This characteristic is different from CTE-NC stage and does not reflect the global burden of CTE pathology. We found that more than half of the decedents (n = 4/6, 66%) had mild levels of p-tau neuronal aggregates around the blood vessel (i.e. <50% of the circumference is occupied by p-tau tangles pathology) and only 2 had moderate-to-severe p-tau neurons aggregates circumference to the blood vessel (i.e. >50% of the circumference is occupied by p-tau tangles pathology). Thus, only 2 decedents were pathologically defined as definite CTE-NC, whereas 4 were considered possible CTE-NC, after expert neuropathologist review.

**Table 2.** Clinicopathological characteristics of participants with definite and possible CTE-NC

Case no.	Age at death, sex	APOE ε4 variant	History of msTBI	Clinical classification	Vascular risk factors or diseases	CTE-related changes	Other pathological changes
1	78 years, male	Yes, APOE ε4	No	Probable AD	None	**Definite CTE-NC** (7 CTE-NC lesions in the superior frontal cortex). p-tau astrocytes around the multiple vessels at depth of sulci and gyral crest in the middle temporal and superior frontal cortices. Subpial ARTAG at gyri and sulci. Patchy distribution of p-tau and NFTs preferentially in superficial cortical laminae	SP ARTAG GM ARTAG WM ARTAG Intermediate ADNC LATE-NC (stage 3) CAA
2	88 years, male	None	Isolated single msTBI	NCI	Diabetes	**Definite CTE-NC** (4 CTE-NC lesions in the superior frontal cortex). p-tau astrocytes around the multiple vessels at depth of sulci and gyral crest in the superior frontal cortex. Subpial ARTAG at sulci. Patchy distribution of tau and NFTs preferentially in superficial cortical laminae	SP ARTAG GM ARTAG WM ARTAG High ADNC LATE-NC (stage 2) CAA
3	71 years, male	None	None	NCI	Hypertension	**Possible CTE-NC** (Single CTE-lesion in the midfrontal cortex. p-tau astrocytes around the single vessel at depth of sulci in the midfrontal cortex. Subpial ARTAG at sulci. Patchy distribution of tau and NFTs preferentially in superficial cortical laminae)	SP ARTAG GM ARTAG WM ARTAG Atherosclerosis
4	84Y, male	Yes, APOE ε4	Isolated single msTBI	Probable AD	Hypertension and diabetes	**Possible CTE** (2 CTE-NC lesions in the superior frontal cortex. p-tau astrocytes around the multiple vessels at depth of sulci in the superior frontal cortex. Patchy distribution of tau and NFTs preferentially in superficial cortical laminae)	GM ARTAG High ADNC LATE-NC (stage 1) PD LBD (neocortical-type) CAA Chronic infarcts
5	93 years, female	None	None	Probable AD	Stroke	**Possible CTE-NC** (2 CTE-NC lesions in the middle temporal cortex. p-tau astrocytes around the multiple vessels in the middle temporal cortex. Patchy distribution of tau and NFTs preferentially in superficial cortical laminae)	GM ARTAG WM ARTAG High ADNC LATE-NC (stage 3) Atherosclerosis Arteriolosclerosis Chronic infarcts
6	91 years, female	Yes, APOE ε4	Yes, isolated single msTBI	NCI	Hypertension	**Possible CTE-NC** (Single CTE-lesion in the middle temporal cortex. p-tau astrocytes around the single vessel at depth of sulci in the middle temporal cortex. Patchy distribution of tau and NFTs preferentially in superficial cortical laminae)	GM ARTAG WM ARTAG Intermediate ADNC CAA Atherosclerosis Chronic infarcts

ADNC, Alzheimer disease neuropathologic change; APOE, apolipoprotein E; ARTAG, aging-related tau astrogliopathy; CAA, cerebral amyloid angiopathy; CTE-NC, chronic traumatic encephalopathy neuropathological changes; LATE-NC, limbic-predominant age-related TDP-43 encephalopathy neuropathologic change; LBs, Lewy bodies; LBD, Lewy body disease; NCI, no cognitive impairment; NFTs, neurofibrillary tangles; PD, Parkinson disease; SD, standard deviation; msTBI, moderate-to-severe traumatic brain injury

We also examined the demographic and clinicopathological characteristics of these 6 CTE-NC participants. The ages of the CTE-NC participants ranged from 78 to 93 years. Three out of 6 participants were diagnosed with dementia. None of the CTE-NC participants had a history of falls, a common cause of brain injury in the elderly. Five CTE-NC participants had either intermediate or high ADNC; the other participant was 1 of 3 with atherosclerosis. Specific clinicopathological information on CTE-NC participants is displayed in Table 2.

### Frequency of CTE-NC supporting features in community-dwelling participants with and without msTBI

CTE-NC supporting features were more common in older community participants with and without msTBI than the CTE-NC diagnostic feature: 22 participants out of 188 (point estimate = 11.8%, 95% confidence interval: 7.2%, 16.4%) and 27 participants out of 188 (point estimate = 14.4%, 95% confidence interval: 9.4%–19.5%) had subpial TSAs in sulci and patchy neurofibrillary tangles in the superficial cortex, respectively. In the participants classified with possible or definite CTE-NC, all 6 brains had patchy neurofibrillary tangles with preferential locations of neurofibrillary tangles in the superficial layer cortex, although only 3 had subpial TSAs in the depth of a sulcus. All 6 CTE-NC brains also had perivascular deposition of p-tau astrocyte pathology within the area of CTE-NC lesions. Furthermore, we also observed p-tau astrocytes deposits around the blood vessel in 2 participants without perivascular p-neuronal tau. Eight participants out of 188 with perivascular astrocytic tau (point estimate = 4.3%, Exact 95% confidence interval: 2.2%, 8.2%), 6 had moderate-to-severe p-tau astrocytes circumference of the blood vessel and only 2 had mild p-tau astrocytes circumference of the blood vessel. Of these 188 participants, only 2 participants had sparse neurofibrillary tangles in the cerebellar dentate nucleus, and neither of these 2 had specific CTE-NC pathologic lesions.

### Frequency of ARTAG in community-dwelling participants with and without msTBI

**Figure 2.**
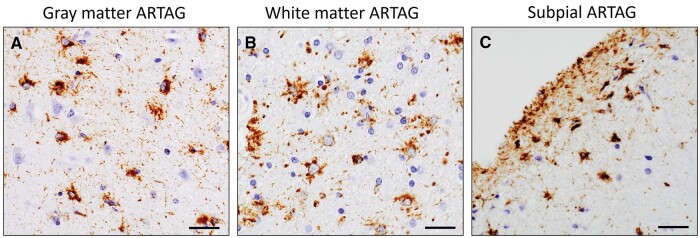
ARTAG pathology in the brain. ARTAG pathology in the gray matter location **(A)**, white matter location **(B)** and subpial location **(C)** of the superior frontal cortex. Scale bars: A–C = 50 μm.

Of these total participants (n = 188), ARTAG (Fig. 2A–G) was found in 143 brains (80%). Of these 143 brains, 137 (77%) had gray matter ARTAG, 97 (54%) had white matter ARTAG, and 92(52%) had subpial ARTAG (Table 1). Approximately three-fourths (n = 131) of decedents had amygdala gray matter ARTAG while more than half of subjects (n = 101) had neocortical gray matter ARTAG in either neocortical region. Of these 101 participants with neocortical gray matter ARTAG, 57 (55%) had gray matter ARTAG in only one cortical region. We also examined the distribution of TSAs and GFAs from gray matter ARTAG of both amygdala and neocortical region and found that TSAs were more common than in GFAs among all 3 regions. For example: of these 131 participants with amygdala gray matter ARTAG, 104 (79%) had TSAs without GFAs, 10 (8%) had GFAs without TSAs, and 17 (13%) had both TSAs and GFAs cell population. A similar distribution of TSAs and GFAs are seen in neocortical gray matter ARTAG. Of these 101 neocortical gray matter ARTAG, 71 (70%) had TSAs without GFAs, 12 (12%) had GFAs without TSAs, and 18 (18%) had both. Likewise, amygdala white matter and subpial ARTAG were also common, present in half of participants but neocortical white matter and subpial ARTAG was found only in a small proportion of participants, ranged from 14% to 16% of participants across regions.

In all regions examined, the presence of any type of ARTAG was strongly associated with having another type of ARTAG in the same region (p < 0.001 for all). For example, the odds of gray matter ARTAG in the amygdala were 3.22 times higher than those with subpial ARTAG in the amygdala (95% CI: [2.27, 4.57], p < 0.001). The odds of gray matter ARTAG in the amygdala were 3.69 times higher than those with ARTAG in the white matter of the amygdala (95% CI: [2.59, 5.26], p < 0.001). Furthermore, the odds of gray matter ARTAG in the anterior temporal pole were 3.29 (95% CI: [1.80, 6.02], p < 0.001) and 4.78 (95% CI: [2.49, 9.19], p < 0.001) times higher than those with subpial ARTAG and white matter ARTAG of the anterior temporal tip, respectively. Similar associations were found between ARTAG types in the superior frontal cortices (p < 0.001 [all 3]).

### Association of msTBI with CTE-NC and ARTAG

**Figure 3.**
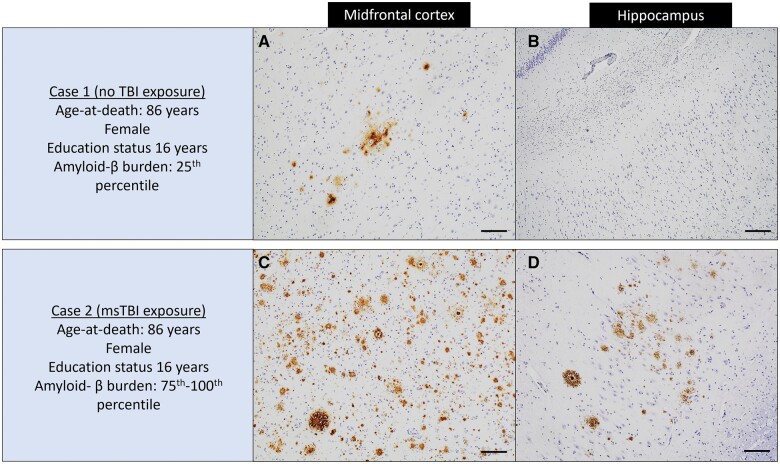
Illustrative cases for amyloid-β pathology. Case 1 is a non-TBI exposure female MCI participant (age-at-death is 86 years and education is 16 years), with amyloid-β burden within the 25th percentile. Case 2 is a msTBI female MCI participant (age-at-death is 86 years and education is 16 years), with amyloid-β burden within the 75th-100th percentile. Both cases were matched one-to-one by age-at-death, sex, education, and dementia status. Images represent 4G8-immunostained sections of the midfrontal (**A, C**) and CA1 subsector of the mid hippocampus (**B, D**). Scale bars: A–D = 200 μm.

In our first set of planned analyses to examine the relationship between msTBI with CTE-NC diagnostic pathology, we found that CTE-NC diagnostic pathology was too infrequent to study discordant pairs across those with and without msTBI. Therefore, no further analytical analyses were performed. Similarly, perivascular astrocytic tau pathology was also too uncommon to examine associations with msTBI. No association was seen between msTBI and CTE-NC supporting pathology (the presence of subpial TSAs or patchy superficial distribution of NFTs) (Table 3). Furthermore, no associations were found between msTBI and any type of ARTAG pathologies (Table 3; Fig. 3).

**Table 3.** Association of msTBI with age-related neuropathologies

Continuous outcomes	Estimates (SE)	p-value
Amyloid-β load, overall	0.339 (0.164)	0.040
PHF-tau tangles density, overall	−0.329 (0.197)	0.096

**Ordinal Outcomes**	**Odds ratio (95% CI)**	**p-value**

Subpial ARTAG at sulci	1.12 (0.45, 2.79)	0.796
Patchy and superficial p-tau tangle	1.45 (0.62, 3.35)	0.383
ARTAG burden, overall	0.79 (0.42, 1.50)	0.485
GM ARTAG burden, overall	0.71 (0.40, 1.25)	0.243
GM ARTAG burden, amygdala	0.71 (0.40, 1.23)	0.226
GM ARTAG burden, neocortex	0.71 (0.42, 1.22)	0.228
WM ARTAG burden, overall	0.89 (0.51, 1.56)	0.694
WM ARTAG burden, amygdala	0.87 (0.50, 1.52)	0.637
WM ARTAG burden, neocortex	0.79 (0.35, 1.75)	0.565
SP ARTAG burden, overall	0.65 (0.36, 1.16)	0.147
SP ARTAG burden, amygdala	0.59 (0.33, 1.06)	0.082
SP ARTAG burden, neocortex	0.92 (0.41, 2.10)	0.859
Neocortical LBs presence	2.35 (0.85, 6.48)	0.097
Pathologic diagnosis of AD	1.15 (0.62, 2.11)	0.643
LATE-NC (stage 2 and above)	0.77 (0.40, 1.48)	0.450
HS	1.00 (0.35, 2.78)	1.00

AD, Alzheimer disease; ARTAG, aging-related tau astrogliopathy; CI, confidence intervals; GM, gray matter; HS, hippocampal sclerosis; LATE-NC, limbic-predominant age-related TDP-43 encephalopathy-neuropathologic change; LBs, Lewy bodies; SD, standard deviation; SP, subpial; WM, white matter.

### Additional analyses

We examined associations of msTBI exposure with several measures of PFH tau, amyloid-β plaques and other neuropathologies. Only one association was seen, with higher amyloid-β load (estimate = 0.339, SE = 0.164, p = 0.040) (Table 3). We ran secondary analyses by replacing the overall amyloid-β variable with diffuse and neuritic amyloid-β plaque variables. No associations were found between msTBI and diffuse and neuritic amyloid-β plaques (p < 0.05; data not shown). There were no associations seen with PHF-tau tangles, pathologic diagnosis of AD, neocortical LBs, LATE-NC, and HS (Table 3). The association between msTBI and tau peptides from the dorsolateral prefrontal cortex in these 188 participants was also explored. Similar to PHF-tau tangles, no associations between msTBI exposure and any of the 5 phosphorylated tau peptides, including S202, T217, S262, S305, and S404 were seen (Table 4).

**Table 4.** Association of msTBI with phosphorylated tau peptides

Phosphorylated tau peptide	Estimates (SE)	p-value
S202	0.099 (0.087)	0.259
T217	−0.131 (0.143)	0.36
S262	−0.098 (0.315)	0.755
S305	0.411 (0.229)	0.076
S404	0.112 (0.118)	0.346

Linear regression models with change in tau phosphorylation stoichiometry as 5 separate outcomes, all adjusted for age-at-death and sex.

## DISCUSSION

In 188 msTBI and matched control participants, we found that CTE-NC is an uncommon pathology in community-dwelling older persons with and without msTBI. This infrequent pathology was seen equally in persons with msTBI and persons without TBI. Pathologic features supporting CTE-NC are more common, but again are not differentially distributed across those with and without msTBI in the community. Finally, although ARTAG is very common in aging, the presence or severity of ARTAG did not differ between msTBI and the matched unexposed groups.

Multiple studies have shown an association between contact sport play, as a proxy for RHI, and CTE-NC, with suggestion of a causal relationship (12–15). CTE-NC has also been described in individuals with a history of RHI from military service, and other activities (40–42). However, the frequency of CTE-NC in individuals with msTBI and the association of CTE-NC with msTBI remain unknown. This study systematically examined CTE-NC pathology from msTBI and matched control participants from the community and found that only a small number of participants (3%) with and without msTBI showed CTE-NC, suggesting that CTE-NC is an infrequent pathology present in community dwelling older persons and can be observed in individuals with and without a history of isolated msTBI. This finding is consistent with the other 3 community-based studies. One of these studies focused on individuals with and without TBI with LOC >1 hour (43) while another included individuals with and without TBI, although the severity of LOC was unspecified (21) Third, a large-scale study found no instances of CTE-NC in cases of mild TBI (20). The infrequent occurrence of CTE-NC in a large community of individuals with a history of single episodes of msTBI is in contrasts to some (6, 44) but not all studies (17, 43). These mixed findings suggest caution should be used when generalizing findings from RHI to single TBI. It is also important to note that none of the CTE-NC participants in the current study had the level of pathologic changes that suggest a high CTE-NC stage, but future consensus meetings are necessary to further refine the staging criteria for CTE-NC, specifically in those with coexisting ADNC pathology (19).

Three other findings are also noted. First, we characterized the CTE-NC cases to as “possible” or “definite” based on the percentage of blood vessels circumference occupied by p-tau tangles to reflect the burden of tau tangles around the blood vessels which is different from CTE-NC stage. We found that only 2 out of 6 CTE-NC participants showed similar level of neuronal tau severity around the blood vessel that than seen in people with a history of repetitive head trauma (19, 45, 46). The remaining 4 CTE-NC individuals do not show the same level of burden of tau tangles around the blood vessels seen in people with history of RHI. In our study, we observe that 2 out of 6 individuals with CTE-NC were female. To date, only a few studies either in contact sports or other settings have shown neuropathological findings of CTE in females (47–49). This could be attributed to historical male dominance in contact sports, where head injuries are prevalent, likely contributes to the notable male bias observed in CTE prevalence thus far (49). Since the prevalence of CTE in females remains uncertain, further research on CTE in females is necessary to gain a better understanding of potential sex-based differences in the risk of CTE-NC and its effects on behavior and cognition. Additionally, we observed that both of these female individuals exhibited CTE-NC lesions in their temporal cortex, in contrast to the other studies that highlight the frontal cortex as the initial region affected in CTE (28, 50, 51). The identification of CTE-NC lesions in the temporal cortex, without corresponding lesions in the frontal cortex, may however be attributed to the sampling method or a lack of examination on both hemispheres. Second, to our knowledge, we are not aware of studies that have systematically examined CTE-supporting features in participants with and without msTBI. This study not only evaluated CTE-NC diagnostic feature, but also examined CTE-NC supporting features and found that CTE-NC supporting features are relatively more common in older individuals in general, compared to CTE-NC diagnostic features, but none of the CTE-NC supporting features were related to the occurrence of msTBI. Finally, this study also examined the perivascular astrocytic tau pathology at the depth of sulci, which was considered a crucial pathology for pathological identification of CTE-NC according to the first consensus criteria (52). However, according to the second consensus criteria (19), perivascular astrocytic pathology deep in sulci is no longer deemed essential for recognition of CTE-NC. Nonetheless, a recent study suggests that the presence of perivascular astroglial p-tau pathology is vital for the best recognition of CTE-NC individuals (53). We found that although perivascular astrocytic p-tau pathology at the depth of sulci is also an infrequent pathology in TBI, it is present both in all of our participants who had CTE-NC lesions (regardless of history of msTBI) and in 2 additional participants without perivascular neuronal tau. This indicates that the presence of p-tau astrocytes may be at least partially related to CTE-NC pathogenesis. Future CTE-NC studies with a specific focus on p-tau astrocytes are required to understand the significance of perivascular astrocytic tau pathology in CTE-NC pathogenesis and to determine whether the presence of perivascular p-tau astrocytic pathology could be considered a diagnostic or supportive feature for the neuropathologic diagnosis of CTE-NC.

This study also provides reference data on the frequency of subpial, gray matter, and white matter ARTAG from the amygdala and neocortical regions and the relation of msTBI with ARTAG. Examination of the occurrence of ARTAG pathology at various sites in the neocortical and amygdala region suggests that ARTAG pathology occurs predominantly in gray matter followed by white matter and subpial sites in both neocortical and amygdala regions. This finding is consistent with other work that suggested a sequential pathological staging for the different ARTAG types (54). The absence of an association between msTBI and wide spectrum of ARTAG pathologies is consistent with other studies (21, 51). Nonetheless, it is worth noting that brain regions such as subcortical and brain stem as well locations such as subependymal and others were not evaluated for ARTAG pathologies. Future study encompassing a wider spectrum of ARTAG pathologies from multiple brain regions will be important to understand the connection between msTBI and ARTAG.

Many clinicopathologic studies have mixed findings concerning the association of TBI with neuropathologic changes. Many studies reporting no association between TBI and AD neuropathologic changes, including CERAD score (55, 56), Thal phase (57), Braak stage (55–57), and AD pathologic diagnosis (55), while others reported higher amyloid-β load (34, 58), and some others (44, 58) reported higher PHF-tau tangles in those with TBI. We found higher levels of amyloid-β in individuals with msTBI, suggesting overlapping pathways between TBI and AD. Prior studies have reported that plaques seen following TBI are typically diffuse over rather than neuritic (58, 59). However, we did not find an association of diffuse plaque with msTBI; this could be because of the small sample size. Further study with large number of samples is required to understand the association of msTBI with diffuse and neuritic plaques. Our study also did not find any association between msTBI and PHF-tau tangles, ARTAG pathology, and phosphorylated tau peptides. The lack of association between msTBI and tauopathies in our study highlights the heterogeneous nature of posttraumatic neuropathology and suggests that TBI-related factors like severity, frequency, and age at injury may play crucial roles in TBI and tau pathology associations. Furthermore, we observed a potential increase in the occurrence of LBs in individuals with msTBI, but the association between msTBI and LBs did not reach statistical significance. This could be because of limited sample size as we have found associations with LBs in our previous studies of TBI (55).

The study has several limitations. First, because msTBI relied on self-report and was solely defined by the duration of LOC, there is a possibility of misclassification for certain participants. Second, there was no information regarding exposure to RHI from contact sport participation, military service, or physical abuse. It is quite possible that brain donors with CTE-NC had RHI exposure, but did not suffer msTBI. Additionally, there was no information on age at msTBI, and the time between msTBI and death, which may influence postmortem study outcomes. Further investigation is needed to compare the relevant exposures observed in our participants with those of subsequent generations to understand how changing exposure levels may impact the risk of neuropathological outcomes. Also, we did not examine participants who report mild TBI or TBI without LOC in this study, nor did we study the relationship with tau pathology specifically ARTAG. Third, all these participants agreed to longitudinal visits and to brain donation, so they are not typical of the general population. Therefore, the frequency of CTE-NC in our community-setting samples may differ from the frequency in other populations. Fourth, given that a majority of our study participants were elderly and many of them exhibited comorbid ADNC, detection of CTE may be difficult and this could result in misclassification. In this study, unequivocal fully circumferential neuronal perivascular tau was seen in only 2 cases and more limited perivascular tau tangles noted in 4 cases with concomitant AD pathology, such that whether we include or exclude perivascular neuronal tau severity characterization, the essential findings of the study remain the same. Finally, given that msTBI can be an asymmetrical disorder and CTE and ARTAG pathology may be present elsewhere in cortical regions, by limiting the examination to one hemisphere and a small number of cortical regions, we may have underrecognized the number of cases with CTE or ARTAG. Despite these limitations, this study has some strengths. The study was conducted using a community-based design, rather than an autopsy series or clinic-based series. Our design is less prone to selection bias. Board-certified neuropathologist and trained examiners who were blinded to TBI status and demographic information performed a uniform neuropathologic evaluation for CTE-NC, ARTAG, and other neuropathologic changes. A systematic evaluation was performed not only for CTE-NC diagnostic feature, but also CTE-NC supporting features.

In summary, this study demonstrates that neuropathological features of CTE are uncommon in the community and do not appear to be associated with msTBI. In addition, the significance of commonly co-occurring perivascular p-tau astrocytes in CTE-NC brains remains uncertain. Finally, ARTAG is a common age-related tauopathy in the community, but we demonstrate no association with msTBI exposure. Further studies are required to determine the relevance of astrocytic tau pathology in aging, CTE, and other dementias.

## Data Availability

Raw data are available by request through the Rush Alzheimer’s Disease Center (RADC) Research Resource Sharing Hub (https://www.radc.rush.edu/).
